# Data for exploring the effect of parameters on decomposition of gas hydrate structure I

**DOI:** 10.1016/j.dib.2018.04.015

**Published:** 2018-04-11

**Authors:** Mohammad Fani Kheshty, Farshad Varaminian, Nafiseh Farhadian

**Affiliations:** a School of Chemical, Gas and Petroleum Engineering, Semnan University, Semnan, I.R., Iran; b Chemical Engineering Department, Faculty of Engineering, Ferdowsi University of Mashhad, I.R., Iran

## Abstract

This article describes initial and final configurations of methane hydrate structure I as PDB file at various cage occupancies and different temperatures. Cage occupancies from full occupancy to 75% at three temperatures of 290 K, 300 K and 310 K are presented. Dissociation behavior of gas hydrate structure I at the temperature of 300 K is shown in changing the potential energy and radial distribution function.

**Specifications Table**

**Table:** 

Subject area	Gas industry
More specific subject area	Molecular dynamics simulation
Type of data	PDB file
How data was acquired	Gromacs Software, VMD software
Data format	Initial structure of gas hydrate, final structure of gas hydrate after simulation at various temperatures
Experimental features	N/A
Data source location	Mashhad/Ferdowsi University of Mashhad/ Simulation and Modeling Research Lab
Data accessibility	Data are presented in PDB files, which is provided as Supplementary data for this article.

**Value of the data**

• The present data will help in construction the methane hydrate gas structure I at various cage occupancies.
• The present data will help in understanding the dissociation behavior of gas hydrate structure when cage occupancy decreases.
• The data will aid the discussion of molecular dynamics simulation parameters changes during the structure dissociation.
• Data presented here provides initial and final condition of simulation at the beginning and end of the simulation at different temperatures.

## 1 Data

The PDB files that are provided as supplementary data for this article show the initial and final structure of methane gas hydrate structure I at various cage occupancies and temperatures (see Supplementary data).

## 2 Materials and methods

### 2.1 Materials

**Fig. 1:**
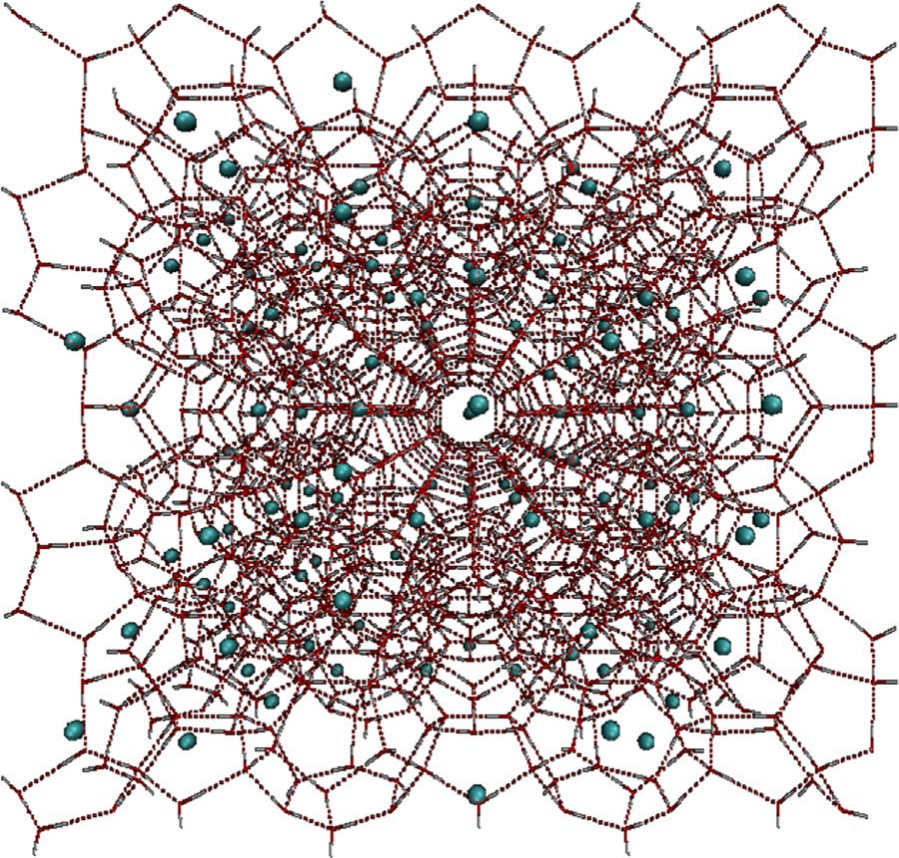
Initial configuration of gas hydrate structure I at the cage occupancy of 80%.

To construct the hydrate structure, the initial positions of oxygen atoms of water molecules were determined using crystallography data obtained from the literature [1], [2]. A box with 3.6 nm × 3.6 nm × 3.6 nm dimensions in x, y and z axes was constructed that included 1242 water molecules in 27 unit cells of hydrate. Fig. 1 shows the initial structure of methane gas hydrate structure I at the 80% cage occupancy.

The PDB files of gas hydrate structure I at various cage occupancies are provided as supplementary data (see Supplementary data).

### 2.2 Methods

MD simulations were performed using Gromacs 4.5.5 package [3] and NPT ensemble was used in all simulations. TIP4P model was applied to simulate water molecules in this study [4]. Methane molecules were assumed as a sphere with zero net charge. The LJ parameters for sphere methane were selected from reference 7 [5] which are needed for simulation. Calculated results were analyzed with MATLAB software.

**Fig. 2:**
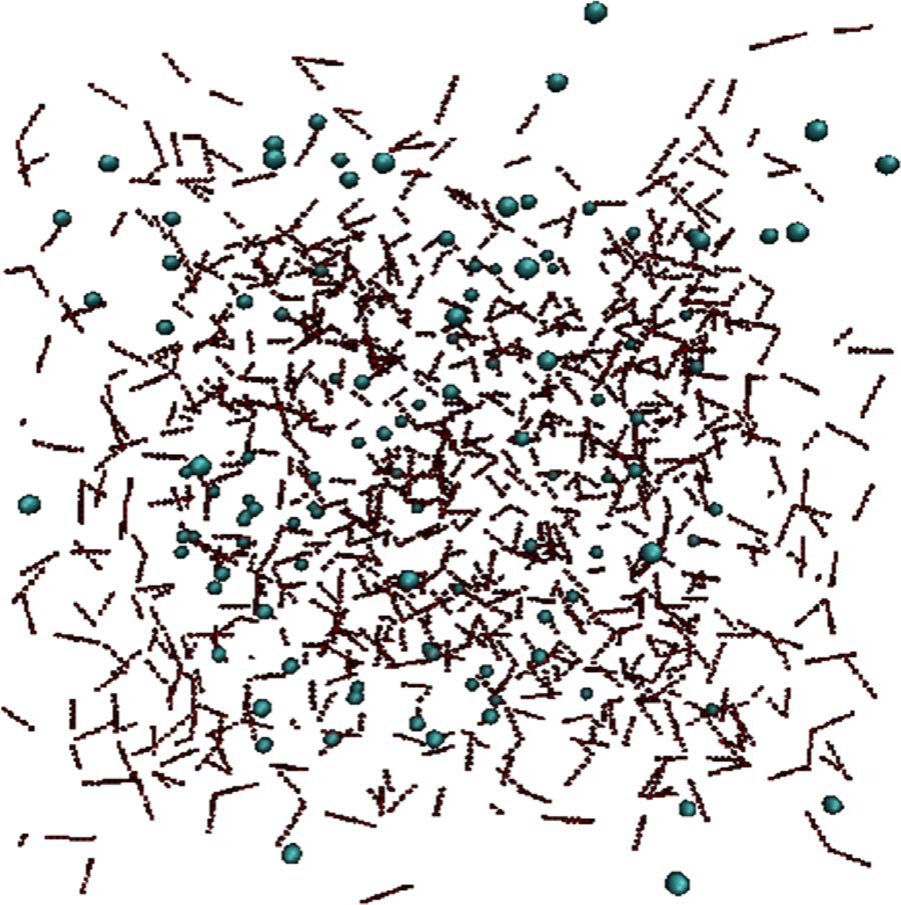
Hydrate structure I at the 80% cage occupancy after 10 ns of the simulation.

**Fig. 3:**
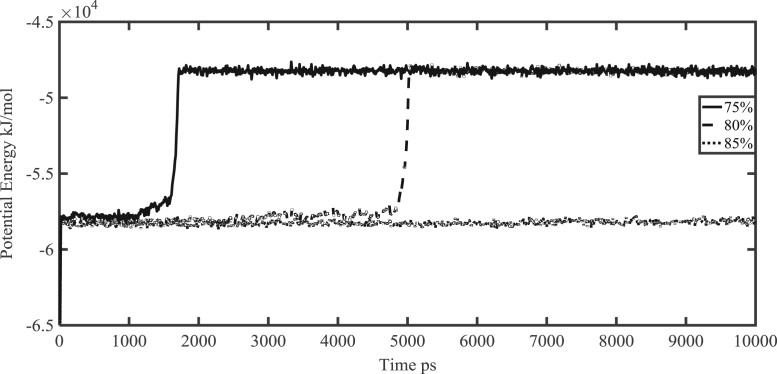
Potential energy at 300 K and three cage occupancies of 75%, 80% and 85%.

The final structure of gas hydrate structure I at the cage occupancy of 80% after 10 ns of the simulation is shown in Fig. 2. Moreover, the potential energy of the systems versus simulation time at various cage occupancies (75%, 80% and 85%) at the temperature of 300 K are shown in Fig. 3.

**Fig. 4:**
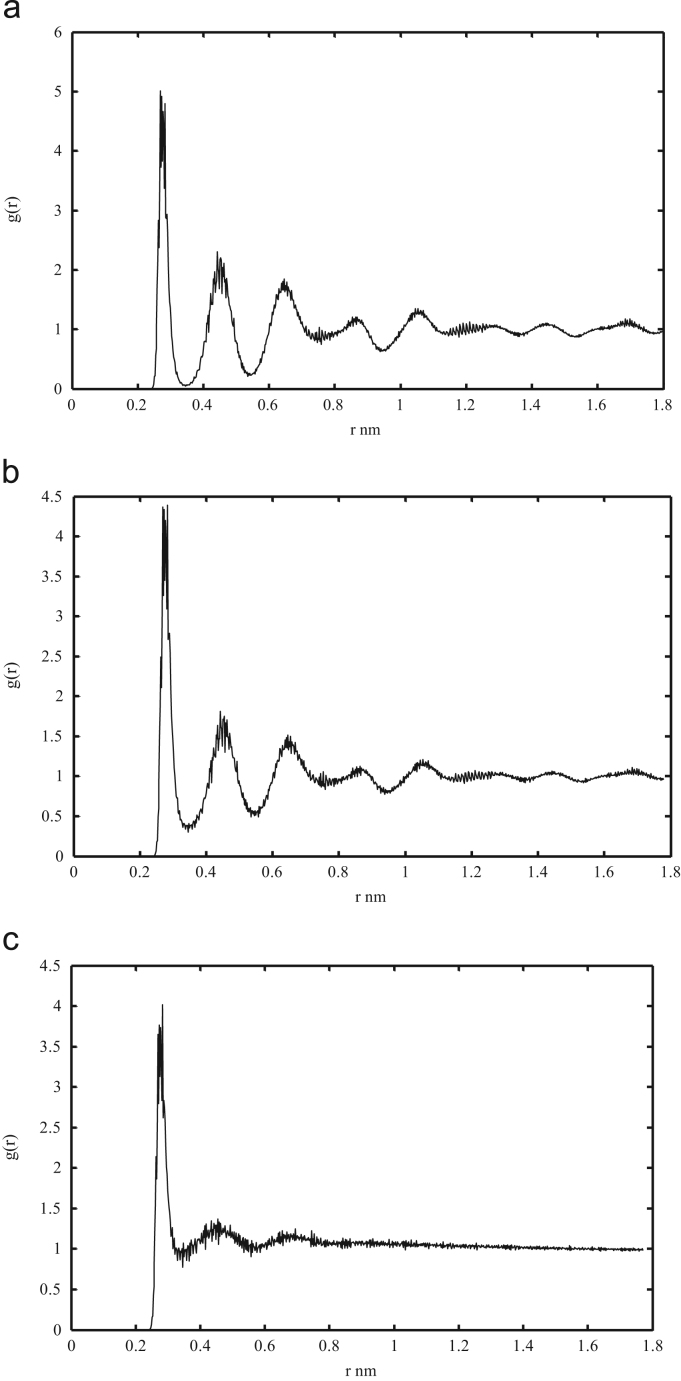
Oxygen-Oxygen RDF at 300 K and 80% cage occupancy after 1 nano second (a), 5 nano second (b) and end of the simulation (c).

Fig. 4 shows the radial distribution function (RDF) of the gas hydrate structure during the MD simulation. Other RDF files as well as initial and final PDB files for each system at different temperatures and various cage occupancies are presented in Supporting data.
