# Supplementary material for: Data for exploring the effect of parameters on decomposition of gas hydrate structure I
Source: Data Brief. 2018 Apr 11;18:1247–51. doi: 10.1016/j.dib.2018.04.015 (PMC5996948; doi:10.1016/j.dib.2018.04.015)
Supplement: Supplementary file 1 — Supplementary material [file mmc1.docx]

**AUTHOR DECLARATION OF INTEREST**

We wish to confirm that there are no known conflicts of interest associated with this
publication and there has been no significant financial support for this work that could have influenced its outcome.

We confirm that the manuscript has been read and approved by all named authors and that
there are no other persons who satisfied the criteria for authorship but are not listed. We
further confirm that the order of authors listed in the manuscript has been approved by all of
us.

We confirm that we have given due consideration to the protection of intellectual property
associated with this work and that there are no impediments to publication, including the
timing of publication, with respect to intellectual property. In so doing we confirm that we
have followed the regulations of our institutions concerning intellectual property.

We understand that the Corresponding Author is the sole contact for the Editorial process
(including Editorial Manager and direct communications with the office). She is
responsible for communicating with the other authors about progress, submissions of
revisions and final approval of proofs. We confirm that we have provided a current, correct
email address which is accessible by the Corresponding Author and which has been
configured to accept email from [n.farhadian@um.ac.ir](mailto:n.farhadian@um.ac.ir).

Signed by all authors as follows:

Mohammad Fani-Kheshty

Farshad Varaminian

Nafiseh Farhadian
